# High abundance of CDC45 inhibits cell proliferation through elevation of HSPA6

**DOI:** 10.1111/cpr.13257

**Published:** 2022-06-01

**Authors:** Yuanyuan Fu, Zhiyi Lv, Deqing Kong, Yuping Fan, Bo Dong

**Affiliations:** ^1^ Sars‐Fang Centre, MoE Key Laboratory of Marine Genetics and Breeding College of Marine Life Sciences, Ocean University of China Qingdao China; ^2^ Institute of Evolution & Marine Biodiversity Ocean University of China Qingdao China; ^3^ Department of Biology Philipps University Marburg Germany; ^4^ Laboratory for Marine Biology and Biotechnology Qingdao National Laboratory for Marine Science and Technology Qingdao China

## Abstract

**Objectives:**

CDC45 is the core component of CMG (CDC45‐MCMs‐GINS) complex that plays important role in the initial step of DNA replication in eukaryotic cells. The expression level of *cdc45* is under the critical control for the accurate cell cycle progression. Loss‐of‐function of *cdc45* has been demonstrated to inhibit cell proliferation and leads to cell death due to the inhibition of DNA replication and G1‐phase arrest. An increasing of CDC45 inhibits cell proliferation as well. Nevertheless, a systematic analysis of the effect of high dose of CDC45 on cell physiology and behaviors is unclear. In the present study, we aimed to investigate the effects and mechanisms of high dose of CDC45 on cell behaviors.

**Materials and Methods:**

We overexpressed *cdc45* in cultured cell lines, *Ciona* and *Drosophila* embryos, respectively. The cell cycle progression was examined by the BrdU incorporation experiment, flow cytometry and PH3 (phospho‐Histone 3) staining. RNA‐sequencing analysis and qRT‐PCR were carried out to screen the affected genes in HeLa cells overexpressing *cdc45*. siRNA‐mediated knockdown was performed to investigate gene functions in HeLa cells overexpressing *cdc45*.

**Results:**

We found that high level of *cdc45* from different species (human, mammal, ascidian, and *Drosophila*) inhibited cell cycle in vitro and in vivo. High dose of CDC45 blocks cells entering into S phase. However, we failed to detect DNA damage and cell apoptosis. We identified *hspa6* was the most upregulated gene in HeLa cells overexpressing *cdc45* via RNA‐seq analysis and qRT‐PCR validation. Overexpression of *Hs‐hspa6* inhibited proliferation rate and DNA replication in HeLa cells, mimicking the phenotype of *cdc45* overexpression. RNAi against *hspa6* partially rescued the cell proliferation defect caused by high dose of CDC45.

**Conclusions:**

Our study suggests that high abundance of CDC45 stops cell cycle. Instead of inducing apoptosis, excessive CDC45 prevents cell entering S phase probably due to promoting *hspa6* expression.

## Graphical abstract

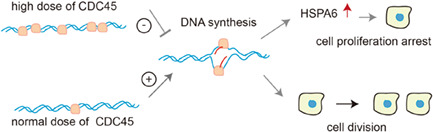

CDC45 is essential for DNA replication. Surprisingly high dose of CDC45 inhibits cell proliferation and blocks cell entering S phase without inducing apoptosis nor aneuploidy as expected. The overexpressed CDC45 induces the elevation of HSPA6, which in turn inhibits cell proliferation.

## 1 INTRODUCTION

The most prominent feature in S phase is DNA synthesis. Accurate DNA replication, in which DNA is copied only once per cell cycle, is important for ensuring the genetic material be passed on to the next generation in a stable manner. The initial step of DNA replication is double strand DNA unwinding by CMG complex.^1^, ^2^, ^3^ CMG complex, which is composed of CDC45 (Cell Division Cycle 45), GINS (Go‐Ichi‐Ni‐San), and MCM2‐7 (Minichromosome Maintenance 2‐7) hexamer, is the key regulator of DNA replication initiation in eukaryotic cells.^2^, ^3^ As the core of DNA helicase, MCM2‐7 hexamer is recruited to the chromatin at G1 phase in an inactivated manner. The binding of CDC45 and GINS to MCMs activates the helicase in S phase and initiate DNA replication.^1^

CDC45 was identified and isolated from budding yeast in 1982.^4^ It has been confirmed that CDC45 binds directly to GINS and MCMs, forming the fully active form of DNA helicase.^5^, ^6^, ^7^, ^8^ Deletion of *cdc45* induces decrease of cell cycle progression in *Leishmania Donovani*.^9^ siRNA‐mediated knockdown of CDC45 could lead to cell proliferation inhibition and cell apoptosis.^10^, ^11^ Downregulation of *cdc45* arrests cells in G1‐phase and represses the expression of G1‐/S‐phase related genes.^1^ Knockdown of *cdc45* inhibits BrdU incorporation into cells, indicating the inhibition of DNA replication.^2^ Degradation of CDC45 impairs DNA synthesis elongation process, leading to the failure of S‐phase.^12^ CDC45 homozygous mutant mice die at early embryonic stage due to the impaired proliferation of inner cell mass.^13^ Dysfunction of CDC45 results in 22q11.2 Deletion Syndrome (also known as DiGeorge Syndrome)^14^, ^15^, ^16^, ^17^, ^18^ and Meier‐Gorlin syndrome.^18^, ^19^, ^20^, ^21^ Patients with 22q11.2 replication syndrome undergo intelligence disability, facial deformity, cardiac defect, and genitourinary abnormalities.^22^

It has been reported that CDC45 degradation in physiological condition depends on ubiquitination and proteasome degradation pathway.^23^ The protein level of CDC45 is important for regulating the ordered activation of origins in S phase.^24^, ^25^, ^26^, ^27^ Low abundance of CDC45 is sufficient to maintain the normal cell cycle progression.^24^ It has been reported that overexpression of *cdc45* inhibits cell proliferation and leads to cell apoptosis,^27^ and vice versa that the CDC45 is not able to be degraded in the apoptotic cells.^23^ However, the mechanism of how expression level of CDC45 affects cell behaviors is unclear.

*Ciona* notochord consists of 40 cells, which have been gone through three complete cell cycle phases. After the completion of cell intercalation, the notochord cells arrange in a single cell profile along anterior‐posterior axis. BrdU incorporation test and PH3 staining reveal that *Ciona* notochord cells are in non‐active cell cycle progression phase.^28^ The notochord cells form a contractile ring in the middle of the cell perpendicular to the anterior‐posterior axis. The activity of the contractile ring, which is reminiscent of cytokinesis ring during mitosis, drives the notochord cells elongating.^29^, ^30^ In order to investigate how the contractile ring assembles, we are particularly interested in the genes highly expressed in notochord cells, as well as involved in cytokinesis. To our surprise, a series of cdc‐related genes including *cdc45*, the irreplaceable DNA replication initiation factor, are highly expressed in the post‐mitotic notochord cells.^28^, ^31^ We wondered whether CDC45 has another function beyond as a DNA replication factor.

Here, we ectopically expressed *cdc45* in the epithelial tissue of *Ciona* and *Drosophila* embryos and cultured cell lines. Agree to the previous report,^27^ cell proliferation was blocked in the cells overexpressing *cdc45*. Yet, we were not able to detect the γH2AX accumulation or cell apoptosis in these cells. We found DNA replication is inhibited in cells overexpressing *cdc45* by BrdU incorporation test and flow cytometry experiment. RNA‐sequencing analysis reveals *hspa6* is the most upregulated gene in HeLa cells overexpressing *cdc45*. The study suggests CDC45 negatively regulates cell cycle through elevation of HSPA6.

## 2 RESULTS

### 2.1 Overexpression of *cdc45* inhibits cell proliferation

**FIGURE 1:**
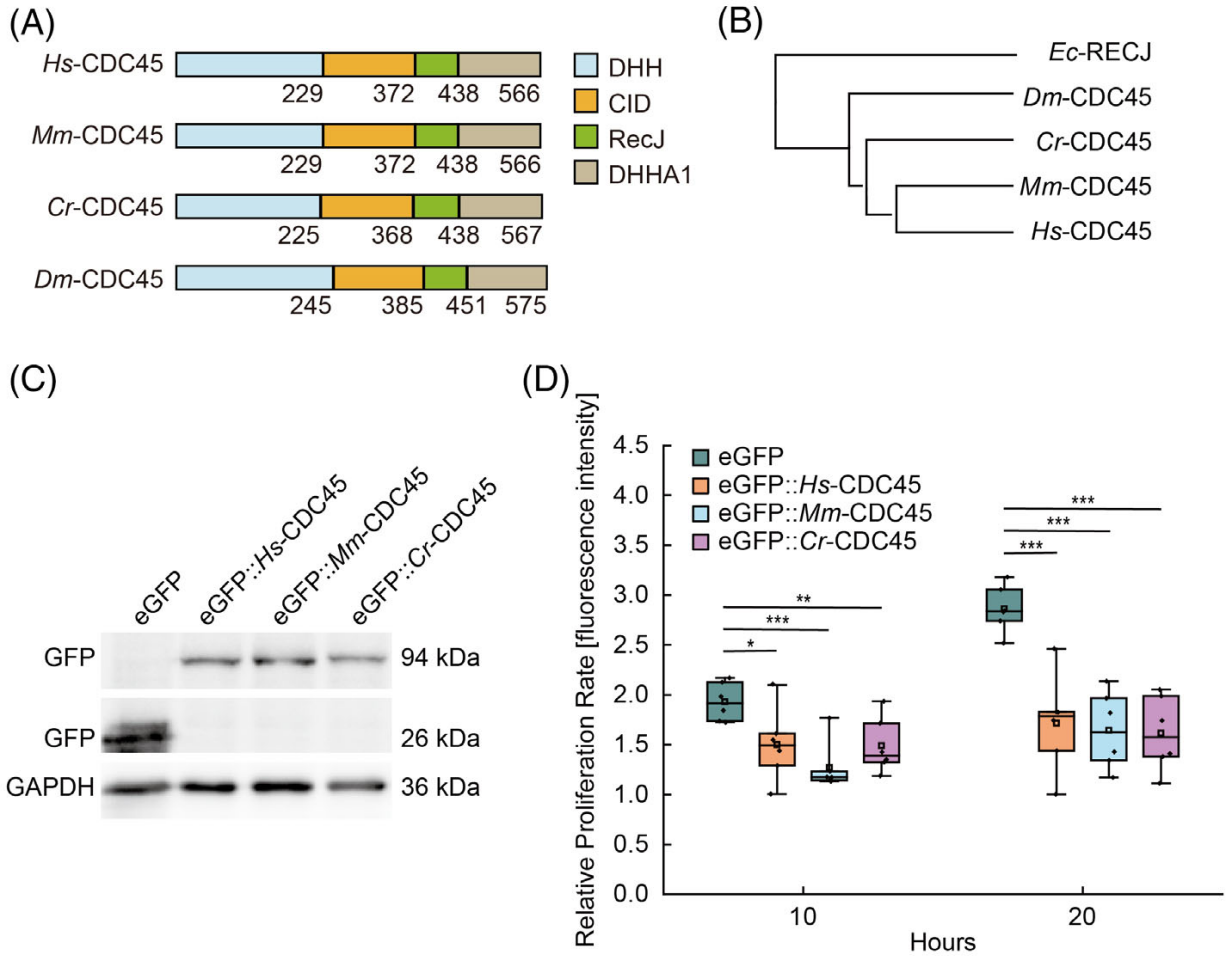
Overexpression of *cdc45* inhibits cell proliferation rate. (A) CDC45 consists of DHH, CID, RecJ, and DHHA1 domains, which are conserved among different species. DHH, Desert Hedgehog protein. CID, C‐terminal domain Interacting Domain. RecJ, 5′‐3′ single strand DNA specific exonuclease. DHHA1, DHH Associated Domain, DHH subfamily 1 members. (B) Phylogenetic tree of CDC45 from eukaryotes and prokaryotes. (C) Western Blot to confirm that eGFP::*Hs‐*CDC45, eGFP::*Mm‐*CDC45, eGFP::*Cr‐*CDC45 was overexpressed in HEK293T cells,respectively. (D) Ectopic expression of *Hs‐cdc45*, *Mm‐cdc45* and *Cr‐cdc45* induced decrease of HEK293T cell proliferation rate, respectively. The images were taken by confocal microscopy at the same position at different time points. Cells containing eGFP::CDC45 only were counted to be defined as cell proliferation according to the fluorescence intensity using the software ImageJ. *n* = 6. *p < 0.05, **p < 0.01, and ***p < 0.001 by Students *t*‐test

CDC45 consists of DHH, CID, RecJ, and DHHA1 domains and is conserved among different species (Figure 1A,B). Knockout of *Hs‐cdc45* in HEK293T cells led to cell proliferation inhibition (Figure S1A–C). In order to investigate the function of *Cr*‐CDC45, we introduced *Cr*‐CDC45 in *Hs‐cdc45* knockout cells. Surprisingly, *Cr*‐CDC45 was not able to restore the cell proliferation. We wondered whether this is a unique feature of *Cr*‐CDC45. To test this idea, we ectopically expressed *Mm*‐*cdc45* in the *Hs‐cdc45* knockout cells. We failed to restore the cell proliferation (Figure S1D). We speculated that the amount of the ectopically expressed *cdc45* excessed, which might lead to the cell proliferation arrest. To confirm this hypothesis, we overexpressed *Hs‐cdc45*, *Mm*‐*cdc45*, and *Cr*‐*cdc45*, which was fused to a N‐terminal enhanced green fluorescent protein (eGFP), respectively in HEK293T cells, and found that the cell proliferation was arrested (Figure 1C,D and Figure S1E). We then examined the protein level of CDC45 by western blot (Figure S2A). The transfection efficiency of HEK293T cells was about 52 ± 2.5% (Figure S2B). We calculated the level of *Hs*‐CDC45 overexpression was 6.2 ± 0.6 times higher compared to the control group according to the result of western blot and transfection efficiency (Figure S2C).

To ensure that the cell cycle arrest is induced by CDC45 specifically, we employed a mutant version, *Cr*‐CDC45‐H44E/Y47A, which interrupts the interaction between CDC45 and PSF1.^5^ As expected, overexpression of *Cr*‐*cdc45*‐H44E/Y47A has no negative effect on cell proliferation (Figure S3A). Considering that CMG complex is composed of CDC45, GINS, and MCM2‐7 hexamer, we were wondering whether it is specific that only *cdc45* overexpression blocks cell proliferation. In addition to *cdc45*, *mcm3*, another component of CMG complex, expresses highly in post‐mitotic *Ciona* notochord cells either.^32^ However, overexpression of either *Hs‐mcm3* or *Cr‐mcm3* has no effect on cell proliferation rate (Figure S3B), indicating it is specific that *cdc45* overexpression blocks cell proliferation.

**FIGURE 2:**
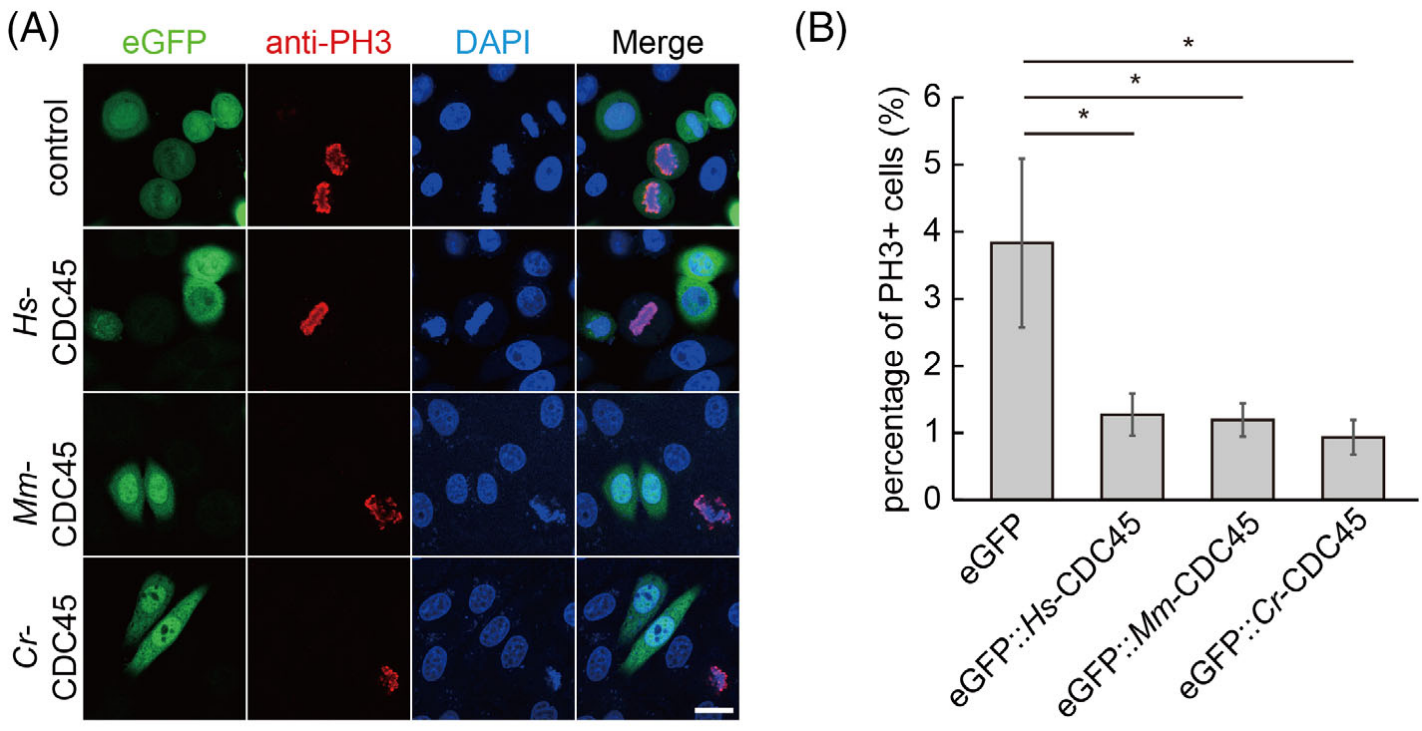
Overexpression of *cdc45* inhibited cells entering into mitosis. (A) Representative images of PH3 staining in HeLa cells overexpressing *cdc45*. Scale bar represents 20 μm. (B) Quantification of the cell proliferation activity, showing the cell number with PH3 positive signals was decreased in HeLa cells overexpressing *Hs‐cdc45*, *Mm‐cdc45* and *Cr‐cdc45*, respectively. One hundred fifty cells per treatment were analyzed. Error bars represent standard deviation. *n* = 3. *p < 0.05 by Students *t*‐test

Next, we detected the mitotic activity of the cells overexpressing *cdc45* by PH3 staining. The percentage of PH3 positive cells was reduced dramatically in GFP‐CDC45 transfected cells (Figure 2A,B). We induced *cdc45*‐HA overexpression in *Drosophila* embryos driven by engrailed promoter, and the amount of PH3 positive cells was reduced in the CDC45‐HA compartments compared to the neighbor ones (Figure S4A,B).

### 2.2 DNA replication is inhibited in the cells overexpressing *cdc45*

**FIGURE 3:**
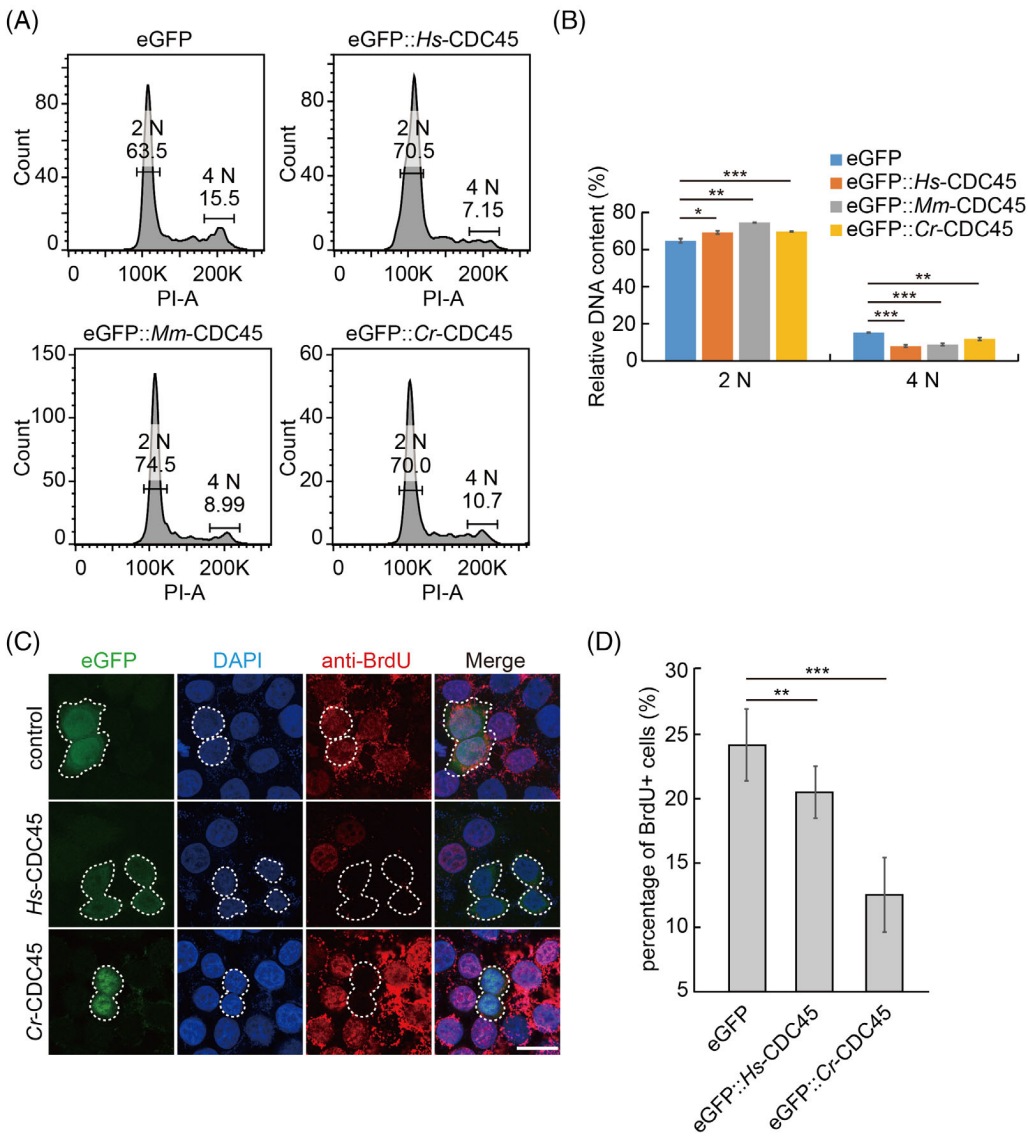
Overexpression of *cdc45* inhibited DNA synthesis. (A) The 2 N population of DNA content was increased in HeLa cells overexpressing *cdc45* compared to the control, whereas 4 N population of DNA content was decreased significantly. (B) Data statistics of panel A. Error bars represent standard deviation. *n* = 3. *p < 0.05, **p < 0.01, and ***p < 0.001 by Students *t*‐test. (C) Representative images of BrdU incorporation of HeLa cells overexpressing *cdc45*. Scale bar represents 20 μm. (D) Ectopic expression of *Hs‐cdc45* and *Cr‐cdc45* induced decrease of BrdU incorporation into HeLa cells, respectively. One hundred fifty cells per treatment were analyzed. Error bars represent standard deviation. *n* = 3. **p < 0.01 and ***p < 0.001 by Students *t*‐test

To gain a deeper insight of how high dose of CDC45 arrests the cell cycle, we analyzed the DNA content profile by flow cytometry. We noticed that the 2 N population was increased, and the 4 N population was decreased correspondingly in the *cdc45* overexpressing cells (Figure 3A,B). Surprisingly, we did not detect the sub‐G1 peak (Figure 3A), the indicator of DNA fragmentation and apoptosis, as expected. This suggested that the high dose of CDC45 arrested the cell cycle before DNA synthesis. We detected the BrdU (Bromodeoxyuridine) incorporation into HeLa cells overexpressing *cdc45*. Ectopic expression of *Hs‐cdc45* or *Cr‐cdc45* induced the decrease of BrdU incorporation into HeLa cells compared to the control, respectively (Figure 3C,D). When we overexpressed *Cr*‐*cdc45*‐H44E/Y47A, the BrdU incorporation rate was comparable with the control group (Figure S3C).

The epidermal cells of *Ciona* embryos undergo active cell division during early embryogenesis. We ectopically expressed *Cr‐cdc45* in the epidermal cells of *Ciona* embryos. Consistent with our previous data, the overlap of BrdU positive cells and *cdc45* overexpressed cells was significantly decreased (Figure S4C,D), indicating DNA replication was inhibited and cell division was blocked.

Taken together, the low incorporation of BrdU into cells overexpressing *cdc45* and flow cytometry assay indicate that high dose of *cdc45* inhibits DNA replication to arrest cells in S phase.

### 2.3 No apoptosis was detected in the cells overexpressing *cdc45*

**FIGURE 4:**
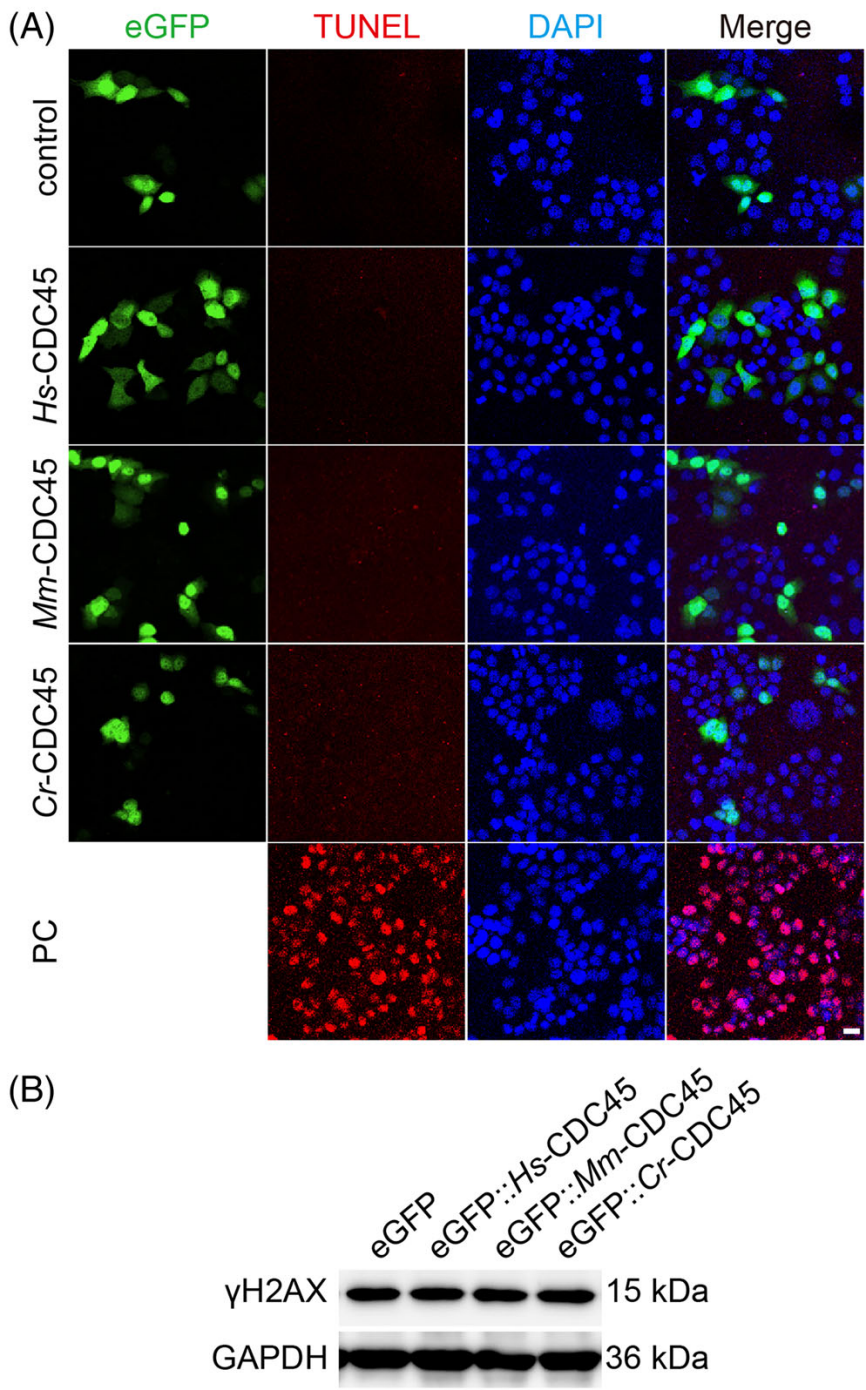
Cell apoptosis and DNA damage detection in HeLa cells overexpressing *cdc45*. (A) TUNEL positive signals were not detected in HeLa cells overexpressing *cdc45*. Scale bar represents 20 μm. PC, Positive Control. (B) Western blot to detect γH2AX accumulation in HeLa cells overexpressing *cdc45* post transfection 48 h. There is no detectable difference among cells overexpressing *Hs‐cdc45*, *Mm‐cdc45*, *Cr‐cdc45*, and control

As mentioned earlier, the sub‐G1 peak was not detected in the *cdc45* overexpressing cells by flow cytometry. To rule out the scenario that high dose of CDC45 leads to cell apoptosis, we conducted the TUNEL assay. However, no difference was observed compared to the control group (Figure 4A).

Considering that the inhibition of DNA replication is related to the DNA damage, we detected the γH2AX accumulation in cells overexpressing *cdc45* by western blot. However, no significant difference was detected among the cells overexpressing GFP‐*cdc45* and GFP alone (Figure 4B and Figure S5A). Moreover, we were not able to detect the unstable ssDNA (single strand DNA) in the cells overexpressing *egfp*‐*cdc45* (Figure S5B).

### 2.4 *hspa6* expresses highly in the cells overexpressing *cdc45*

To determine the mechanism of the high abundance of CDC45 leading to cell cycle arrest, we did RNA‐seq to screen the gene expression profile in HeLa cells overexpressing *Hs‐cdc45*, *Mm‐cdc45*, and *Cr‐cdc45*, respectively.

**FIGURE 5:**
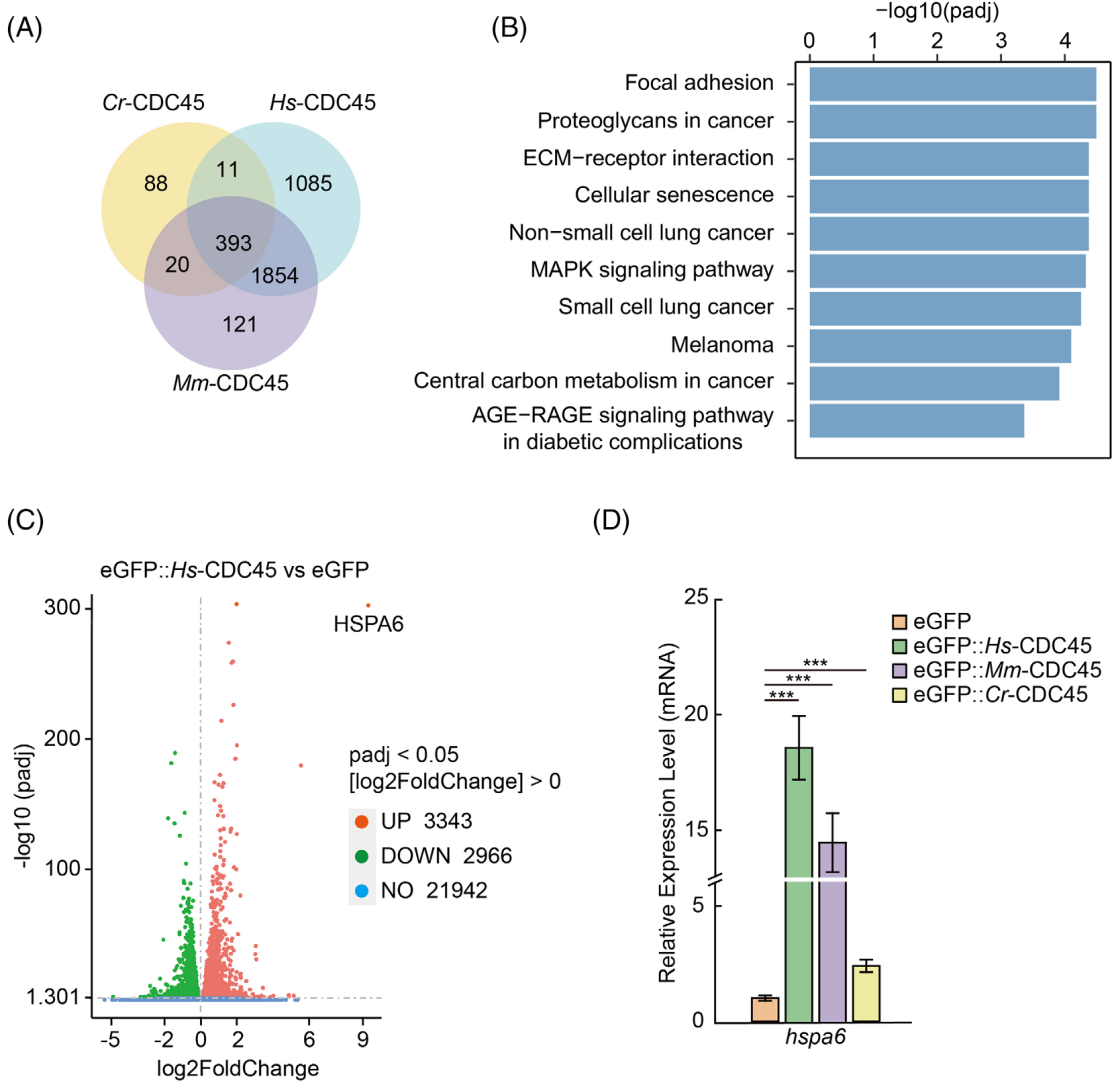
*hspa6* is the downstream gene regulated by *cdc45* overexpression in HeLa cells. (A) Venn map of up‐regulated genes in HeLa cells overexpressing *cdc45* from RNA‐sequencing (RNA‐seq) data. (B) KEGG enrichment analysis of up‐regulated genes shared in HeLa cells overexpressing *Hs*‐*cdc45*, *Mm‐cdc45*, and *Cr‐cdc45* (top 10 terms of functional pathways). KEGG enrichment analysis showed cellular senescence has been enriched as a highly ranked term. (C) Volcano plot of differentially expressed genes in HeLa cells overexpressing *Hs‐cdc45. hspa6* was the most up‐regulated gene in HeLa cells overexpressing *cdc45*. (D) qPCR to confirm that mRNA level of *hspa6* was increased in HeLa cells overexpressing *Hs‐cdc45*, *Mm‐cdc45*, and *Cr‐cdc45* compared to the control group, respectively. Error bars represent standard deviation. *n* = 3. ***p < 0.001 by Students *t*‐test

We sorted 2388, 3343, and 512 up‐regulated genes in cells overexpressing *Hs‐cdc45*, *Mm‐cdc45*, and *Cr‐cdc45* compared to the cells transfected with empty vector, respectively (Figure 5A). Three hundred and ninety‐three genes were in the intersect between these datasets. KEGG enrichment analysis of up‐regulated genes shared in HeLa cells overexpressing *Hs‐cdc45*, *Mm‐cdc45*, and *Cr‐cdc45* showed cellular senescence has been enriched as a highly ranked term (Figure 5B), indicating the cell cycle arrest induced by expression of CDC45 may lead to cell senescence. Among these up‐regulated genes, *heat shock 70 kDa protein 6* (*hspa6*) was the most up‐regulated gene (Figure 5C and Figure S6B). We validated the expression profile of *hspa6* by qPCR (Figure 5D).

### 2.5 Down‐regulating *hspa6* partially restored the proliferation of the cells overexpressing *cdc45*

**FIGURE 6:**
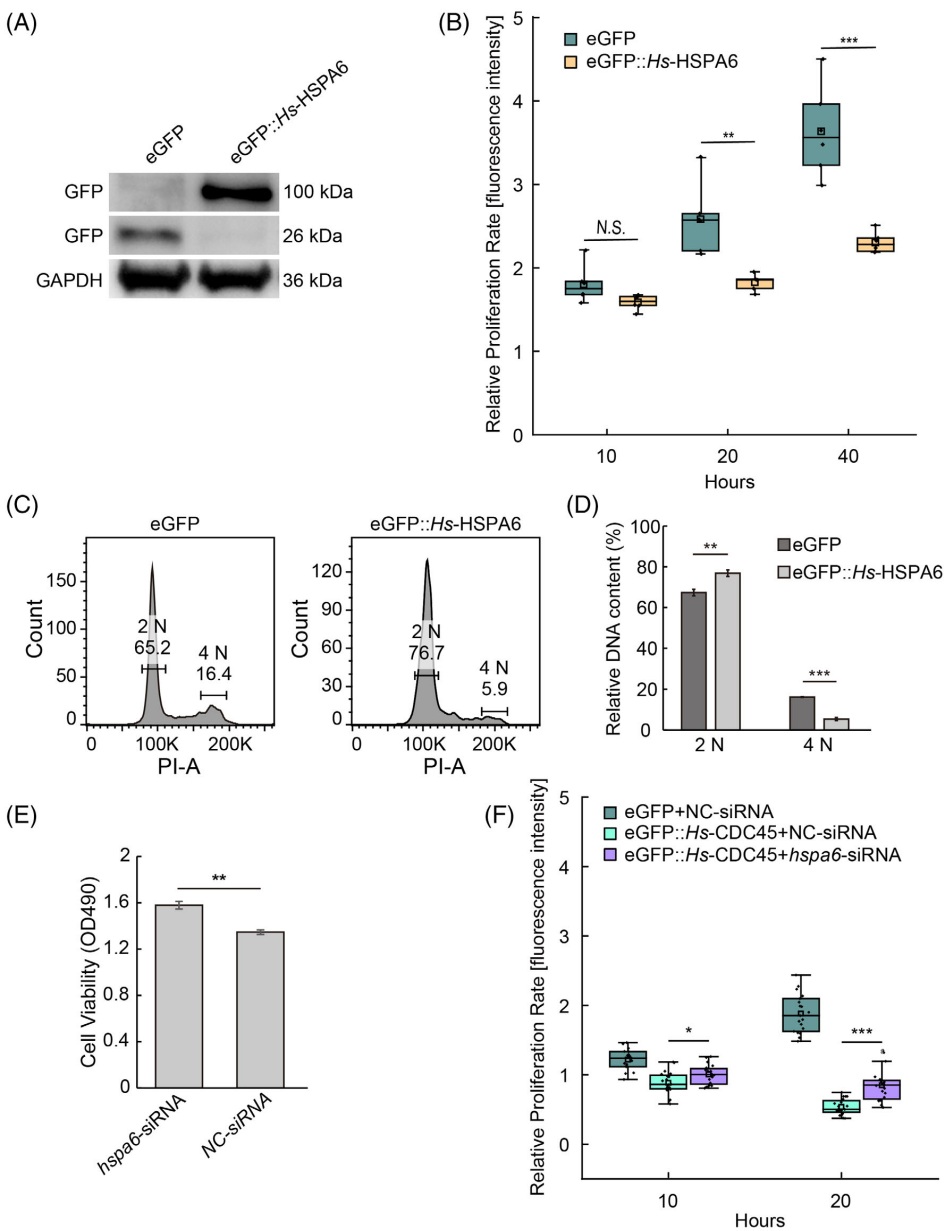
High level of CDC45 inhibits cell cycle through elevation of *hspa6*. (A) Western Blot to confirm eGFP::*Hs‐*HSPA6 was overexpressed in HeLa cells. (B) Ectopic expression of *hspa6* induced decrease of proliferation rate of HeLa cells. N.S., no significant. *n* = 6. N.S., **p < 0.01, and ***p < 0.001 by Students *t*‐test. (C) The 2 N population of DNA content was increased in HeLa cells overexpressing *hspa6* compared to the control, whereas 4 N population of DNA content was decreased. (D) Data statistics of panel C. Error bars represent standard deviation. *n* = 3. **p < 0.01 and ***p < 0.001 by Students *t*‐test. (E) Transfection of *hspa6*‐siRNA promotes HeLa cells proliferation rate post transfection 48 h detected by MTT assay. NC, negative control. Error bars represent standard deviation. *n* = 3. **p < 0.01 by Students *t*‐test. (F) Transfection of siRNA of *hspa6* partially rescued the cell proliferation inhibition phenotype induced by *Hs‐cdc45* overexpression in HeLa cells. *n* = 20. *p < 0.05 and ***p < 0.001 by Students *t*‐test

We overexpressed *hspa6* fused to a N‐terminal eGFP in cultured cell line and found the high level of HSPA6 inhibited HeLa cell proliferation, mimicking *cdc45* overexpression phenotype (Figure 6A,B). Next, we preformed flow cytometer analysis. The 2 N population was increased and the 4 N population was decreased (Figure 6C,D), similar with the cells overexpressing *cdc45*.

siRNA against *hspa6* in normal HeLa cells promotes cell proliferation rate detected by MTT assay post transfection 48 h (Figure 6E). We asked if *hsap6* is involved in the inhibition of cell cycle induced by high dose of CDC45. To confirm this, we applied siRNA against *hsap6* in HeLa cells overexpressing *Hs‐cdc45*. Strikingly, the cell proliferation defect caused by high dose of CDC45 was partially rescued by *hspa6* knockdown (Figure 6F). Taken together, the data suggests high dose of CDC45 blocks cell cycle by regulating *hspa6* expression, rather than inducing cell apoptosis.

## 3 DISCUSSION

CDC45 is an irreplaceable factor to initiate DNA replication in eukaryotic cells. As the key component of CMG complex to unwind double strand DNA, CDC45 is conserved among different species. Deletion or dysfunction of CDC45 leads to cell proliferation inhibition even cell death.^9^, ^10^, ^11^ Here, we firstly confirmed knockout of *cdc45* induced by CRISPR/Cas9 inhibits HEK293T cell proliferation. This gives evidence that CDC45 is important for cell cycle progression.

There are abundant potential replication origins on chromosome, bound by inactive MCM complex. The CDC45 binding to MCM complex triggers the origin firing. Elevated expression of *cdc45* induces DNA replication stress, abnormal cell cycle progression even tumorigenesis.^25^, ^26^, ^33^, ^34^, ^35^, ^36^, ^37^, ^38^ Our study systematically investigated CDC45 from different species, and characterized the cell proliferation defects induced by high level of CDC45 using BrdU incorporation assay, PH3 staining, and flow cytometry. Meanwhile, overexpression of *Cr*‐*cdc45*‐H44E/Y47A, the mutant version that has been confirmed to affect the interaction between CDC45 and PSF1,^5^ has no negative effect on cell proliferation rate and BrdU incorporation rate. *cdc45* expresses highly in post‐mitotic *Ciona* notochord cells with no DNA replication.^28^, ^31^ CDC45 binds to GINS complex through PSF1. Disruption of the interaction between CDC45 and GINS complex affects DNA synthesis in *Xenopus* extracts, indicating the interaction between CDC45 and GINS complex is essential for DNA replication.^5^ Ectopic expression of *Cr*‐CDC45‐H44E/Y47A promotes cell proliferation slightly instead of inhibiting cell growth may results from the mutant binds to MCM2‐7 hexamer competitively to affect the assemble of CMG complex, indicating low level of CDC45 promotes cell growth. However, our result showed that deletion of *cdc45* inhibits cell proliferation rate.

The results indicate that high level of CDC45 is critical to maintain the homeostasis of *Ciona* notochord cells. However, overexpression of *mcm3*, another component of CMG complex, which expresses highly in post‐mitotic *Ciona* notochord cells either,^32^ has no effect on cell growth. The number of MCM2‐7 hexamer bound to chromatin exceeds greatly to ORC complex bound to origins.^39^, ^40^ The activation of MCM2‐7 hexamer induced by recruitment of CDC45 to the origins is non‐synchronous at S phase.^41^ Not all chromatin‐bound MCM2‐7 hexamer could be activated by CDC45 at the initial step of DNA replication,^41^ indicating the low level of CDC45 is essential for accurate DNA replication, whereas the level of MCM complex is not so important as CDC45 does. The result indicates the specificity of *cdc45* overexpression on cell cycle inhibition.

It has been reported that overexpression of *cdc45* led to the standstill and collapse of replication forks, thereafter the DNA damage responses with the accumulation of phospho‐ATM, phospho‐ATR, and γH2AX.^26^, ^27^, ^37^ Elevated expression level of SLD3, SLD7, and CDC45 activates late origins firing in early S phase since the origins firing is nonsynchronous in S phase.^22^ High expression of *cdc25b* or *c‐myc* promotes the binding of CDC45 to the chromatin, which leads to the activation of replication origins untimely, replication fork stall or collapse and DNA damage.^25^, ^26^ Inhibition of the activity of CHK1 results in the excess binding of CDC45 to replication origins, thereafter the cell apoptosis.^34^, ^35^ However, we failed to detect γH2AX accumulation and TUNEL positive signals in the cells overexpressing *cdc45*, albeit high expression of *cdc45* leads to HeLa cell death in the end. The reason for this inconsistence might be the dosage of *cdc45* varies in the transfected cells, or the molecular function of CDC45 is much more complicated than our expectation. KEGG enrichment analysis revealed that cellular senescence has been enriched as a highly ranked term in HeLa cells overexpressing *cdc45*, indicating the cell cycle arrest induced by *cdc45* overexpression may due to the cell senescence instead of cell apoptosis.

Using transcriptome sequence and qRT‐PCR confirmation, we identified *hspa6* expresses highly in HeLa cells overexpressing *cdc45*. Overexpression of *hspa6* inhibits cell proliferation rate, mimicking the *cdc45* overexpression phenotype.

HSPA6 belongs to HSP70 family. Compared to other members of HSP70 family, the function of HSPA6 is less known. It has been reported that co‐application of celastrol and arimoclomol induces expression of heat shock proteins to assist differentiated neurons to get through stress, the protection of which was inhibited by HSPA6 silencing.^42^ In addition to stress response protection, the expression level of HSPA6 is closely correlated to multi tumorigenesis.^43^, ^44^, ^45^, ^46^ The survival rate of patients with breast cancer is positively related to the expression level of HSPA6.^47^ Overexpression of *hspa6* inhibits proliferation, migration and invasion of Triple‐Negative Breast Cancer cells.^47^ Up‐regulation of *hspa6* inhibits Smooth Muscle Cell growth either.^48^ Our result shows that high expression of *hspa6* inhibits HeLa cells proliferation rate, which is in agreement with the previous reports.

Under heat shock, HSPA6 localizes on the nuclear speckles, which are the sites of transcription factories on the genome.^49^ Upon the treatment of Triptolide, an RNA transcription inhibitor, the nuclear speckle localization of HSPA6 disappears.^50^ The upregulation of *hspa6* induced by *cdc45* overexpression may cause the slowing cell cycle. The proteins from aggregates when the cells are treated by the proteotoxic stress‐inducing agent MG132. It has been reported HSPA6 localizes to the periphery of the protein aggregates.^51^ In addition, HSPA6 has been confirmed to enhance the effect of garlic extract‐induced signaling cascade of ATM‐CHK2‐CDC25C‐p21WAF1‐CDC2 and phosphorylation of MAPK and AKT signaling to arrest bladder cancer EJ cell to G2/M phase,^52^ implying the closely correlation between HSPA6 and cell cycle progression. KEGG analysis revealed HSPA6 belongs to MAPK pathway, which has been confirmed to correlate closely to cell cycle regulation.^53^

Taken together, our study suggests that instead of inducing apoptosis, high level of CDC45 blocks cell cycle through upregulating HSPA6. It is possible that besides being a key component during DNA unwinding, CDC45 functions as a cell cycle regulator, which might be a potential therapeutic target for cancer disease.

## 4 MATERIALS AND METHODS

### 4.1 Plasmid construction

The *Hs‐cdc45*, *Mm‐cdc45*, *Cr‐cdc45*, *Hs‐mcm3*, *Cr‐mcm3* sequence were searched in NCBI (gene ID are listed in Table S1). The putative ORF of *Hs‐cdc45*, *Mm‐cdc45*, *Cr‐cdc45*, *Hs‐mcm3* and *Cr‐mcm3* were identified and amplified by PCR using cDNA from HeLa cells, *Mouse* and *C. robusta* respectively. Then, *Mm‐cdc45* and *Cr‐cdc45* were subcloned into the vector pEGFP‐C1 (Clontech) with the enzyme digestion site Xho I and BamH I. *Hs‐cdc45* was subcloned into the vector pEGFP‐C1 (Clontech) with the enzyme digestion site EcoR I and Sma I. *Hs‐mcm3* and *Cr‐mcm3* was subcloned into the vector pEGFP‐N1 (Clontech) with the enzyme digestion site Nhe I and Sma I.

The 2 kb DNA sequence upstream of Epi I from *C. savignyi* was amplified by PCR and subcloned into the empty vector pEGFP‐1 (Clontech) to generate the plasmid *Cs‐epi I* > eGFP expressing specifically in *Ciona* epidermis cells. The restriction sites were Xho I and Xma I. Thereafter the eGFP sequence was replaced by eGFP::*Cr*‐CDC45 from the plasmid CMV>eGFP::*Cr*‐CDC45 with the restriction sites Xho I and Not I to generate the plasmid *Cs*‐*epi I*>eGFP::*Cr*‐CDC45.

The *Hs‐hspa6*, was searched in NCBI (gene ID are listed in Table S1). The putative ORF of *Hs‐hspa6* was identified and amplified by PCR using cDNA from HeLa cells overexpressing of *Hs‐cdc45*. Then, *Hs‐hspa6* was subcloned into the vector pEGFP‐C1 (Clontech) with the enzyme digestion site Sal I and Kpn I.

The gRNA sequence of *Hs‐cdc45* (TATATTGCACGTGGTCACAC) we used was followed McKinley.^54^ The sequence was synthesized and subcloned into the empty vector p2U6‐pCAG‐Cas9‐puro to generate the plasmid p2U6‐pCAG‐Cas9‐puro‐*Hs‐cdc45*‐sgRNA.

All PCR primers are listed in Table S2.

### 4.2 Cell culture

HeLa and HEK293T cells were cultured in complete medium supplemented with 10% fetal bovine serum (BI) at 37°C, with 5% CO_2_ and 95% relative humidity as described previously.^55^ Transfection of plasmids into cultured cells was accorded to the manufacturer's instructions (Lipo3000 from Thermo).

### 4.3 Knockout and rescue experiment

A single guide RNA (sgRNA) target for *Hs‐cdc45* was designed at the third exon of *cdc45* genome sequence which is located onto the N terminal of its coding sequence.^54^ To determine whether the sgRNA worked or not, we used T7 endonuclease I that recognizes and cleaves non‐perfectly matched DNA. The DNA was extracted from HEK293T cells post‐transfected with plasmid p2U6‐pCAG‐Cas9‐puro‐*Hs‐cdc45*‐sgRNA 48 h. Primers were designed at both ends of the target recognized by *Hs‐cdc45‐sgRNA* for about 400 bp. Two bands (150 and 250 bp) cleaved by T7 endonuclease I and sequencing result showed that the sgRNA targeted for *Hs‐cdc45* worked.

As for the rescue experiment, the plasmids pEGFP‐C1 and p2U6‐pCAG‐Cas9‐puro‐*Hs‐cdc45*‐sgRNA were co‐transfected into HEK293T cells to be determined as the control. The plasmids p2U6‐pCAG‐Cas9‐puro‐*Hs‐cdc45*‐sgRNA and CMV>eGFP::*Mm*‐CDC45 or CMV>eGFP::*Cr*‐CDC45 were co‐transfected into HEK293T cells to be determined as the *Hs‐cdc45* knockout and rescued by ectopic expression of *cdc45*.

### 4.4 RNAi

The siRNA of *hspa6* was designed and synthesized by GenePharma company (listed in Table S2). As for the rescue experiment, the plasmid CMV>eGFP::*Hs*‐CDC45 was co‐transfected with siRNA of *hspa6* into HeLa cells.

### 4.5 Cell proliferation assay

For cell proliferation detection, HEK293T and HeLa cells were plated in 24‐well, and were transfected by the designated plasmids using Lipofectamine® 3000. The cells transfected successfully were marked by GFP. The images taken by Nikon A1 confocal microscopy at 488 nm channel was used to acquire the images at the same position of the 24‐well plate at different time points. ImageJ (https://imagej.nih.gov/ij/download.html) was used to measure the total area of GFP positive cells. The cell proliferation rates were derived by the changes of the area of GFP positive cells over time. The formula is as follows:

Relative Proliferation Rate [fluorescence intensity] = *A*_0_/*A*

*A*: Total area of cells containing fluorescence at definite time after transfection.

*A*_0_: Total area of cells containing fluorescence at time 0 was defined for different experiments.

We defined cells post transfection 20 h as time 0.

For MTT assay, cells were incubated in MTT solution at a final concentration of 0.5 mg/ml in DMEM for 4 h at 37°C. DMSO was added to dissolve the formazan crystal. The value of optimal density was measured at 490 nm using a microplate reader (GENios, TECAN).

### 4.6 BrdU incorporation

*Ciona* embryo electroporated with *Cs‐epi I* > eGFP::*Cr*‐CDC45 were cultured in sea water containing 5 mM 5‐bromo‐2′‐deoxyuridine (BrdU, B23151, Invitrogen) at 11.5 hpf for 2 h at 16°C. The protocol to detect the BrdU incorporation followed Lu.^56^

HEK293T and HeLa cells overexpressing of *cdc45* were cultured in complete medium containing 10 μM BrdU at post‐transfection 24 h for 8 h, and quickly fixed by 4% formaldehyde in PBS for 2 h at RT. The protocol to detect the BrdU incorporation was accorded to the manufacturer's instructions (Mouse anti‐BrdU from Invitrogen).

### 4.7 TUNEL staining

HEK293T and HeLa cells overexpressing of *cdc45* were quickly fixed by 4% formaldehyde in PBS for 2 h at RT. Thereafter TUNEL staining to detect cell apoptosis was accorded to the manufacturer's instructions (TUNEL BrightRed Apoptosis Detection Kit from Vazyme).

### 4.8 PH3 staining

HeLa cells overexpressing of *cdc45* were quickly fixed by 4% formaldehyde in PBS for 2 h at RT. The fixed cells were rinsed with PBS for three times, thereafter three times of washing by PBST (PBS with 0.1% TritonX‐100), each time is 10 minutes. Then the fixed cells were stained with the primary antibody of PH3 gifted from Gao's lab for 16 h at 4 °C after blocked by 10% goat serum dissolved in PBST. After three times washing with PBS, the cells were then incubated with the secondary antibody Alexa Fluor 568 anti‐Rabbit IgG (Table S3) for 2 h at room temperature. The cells were mounted using Vectashield (Vector Laboratories) mounting medium with DAPI (Table S3) and stored at 4°C.

### 4.9 ssDNA detection

Visualizing of unstable ssDNA of HEK293T cells overexpressing of *cdc45* was performed as described.^27^, ^57^ HEK293T cells were cultured in complete medium containing 10 μM BrdU for 24 h before transfection. Cells were fixed quickly by 4% formaldehyde in PBS at post‐transfection 24 h for 2 h at RT. The protocol to detect the BrdU incorporation was accorded to the manufacturer's instructions (Mouse anti‐BrdU from Invitrogen) without the step of dsDNA denaturing with HCl.

### 4.10 Western blot analysis

Cells were lysed directly in NP40 buffer. Proteins were separated SDS‐polyacrylamide gel electrophoresis and transferred onto a PVDF membrane (Merck Millipore). Membranes were blocked by 6% skimmed milk dissolved in Tris‐buffered saline with Tween 20 (TBST, 10 mM Tris–HCl, pH 7.5, 150 mM NaCl and 0.5% Tween 20) for at least 1 h at room temperature, followed by incubation with the primary antibody (Table S3) for at least 16 h at 4°C. After washing three times with TBST, membranes were incubated with the horseradish peroxidase–conjugated secondary antibody (Table S3) for 2 h at room temperature. Immunoblots were developed with film exposure (ECL reagent from Vazyme). All original western blot data was presented in Figure S7.

### 4.11 Flow cytometry

HeLa cells overexpressing of *cdc45* post‐transfection 48 h were trypsinized, washed with PBS for one time. PI staining to detect DNA content was then accorded to the manufacturer's instructions (Cell Cycle and Apoptosis Analysis Kit from Beyotime). Flow cytometry was then performed with a BD FACS Aria III flow cytometer equipped with blue and green lasers. Data was analyzed using the software FlowJo (FlowJo, LLC).

### 4.12 RNA extraction, RNA sequencing and data analysis

The total RNA was extracted from HeLa cells overexpressing of eGFP, eGFP::*Hs*‐CDC45, eGFP::*Mm*‐CDC45, eGFP::*Cr‐*CDC45 post‐transfection 48 h using RNAiso plus reagent (Takara). The integrity and quality of total RNA was determined by 1% agarose gel electrophoresis and Nanodrop spectrophotometry (Eppendorf). Each sample collection was repeated three times. Transcriptome sequencing was performed by Novogene. The library was constructed followed NEB common library construction method.^58^ Illumina sequencing was operated after qualified of examination in depot. The clean reads were obtained for subsequent analysis after filtration of original data, error rate examination of sequencing and examination of GC content distribution. The clean reads were then compared to the reference genome. Differential gene enrichment was analyzed using NovoMagic (https://magic.novogene.com/).

### 4.13 qRT‐PCR analysis

The total RNA was extracted from HeLa cells overexpressing of eGFP, eGFP::*Hs*‐CDC45, eGFP::*Mm*‐CDC45, eGFP::*Cr‐*CDC45, eGFP::*Cr*‐CDC45‐R406A post‐transfection 48 h using RNAiso plus reagent (Takara). The integrity and quality of total RNA was determined by 1% agarose gel electrophoresis and Nanodrop spectrophotometry (Eppendorf). The first‐strand cDNA was synthesized using 1 μg total RNA per 20 μl reaction system by reverse transcriptase (Vazyme). The primers of RT‐qPCR were designed in the website PrimerBank (https://pga.mgh.harvard.edu/primerbank/index.html). RT‐qPCR was performed using the SYBR Green PCR Master Mix (Vazyme) on Light Cycler 480 (Roche). All RT‐qPCR primers were listed in Table S2. *Hs‐β‐actin* was used as the reference gene. Data was calculated using 2‐ΔΔCt method.

### 4.14 Ciona and *Drosophila*

*Ciona* used in this study was collected from Qingdao and Rongcheng harbor bay (Shandong Province, China). The animals were then maintained in the seawater with aeration and illumination in our laboratory. The adult animals were dissected by scissors to collect gametes before fertilization. To get trans‐gene *Ciona* embryos, the fertilized eggs (300 μl) were mixed with 60 μg of plasmids in 122 μl final volume adjusted with water and was electroporated after adding 378 μl of 0.72 M D‐mannitol using a Gene Pulser Xcell System (BIO‐RAD) in 0.4 cm cuvettes after dechorionation. The exponential protocol was used with 50 V and 1500 μF as a parameter. Once electroporated, the fertilized eggs were washed, and cultured at 16°C.

Fly stocks used were engrailed‐Gal4 (Bloomington Drosophila Stock Center, No.: 30564) and UAS‐cdc45‐HA (FlyORF: F000766). All crosses and cages were kept at 25°C.

### 4.15 Confocal imaging and data analysis

Images were taken using Nikon A1 confocal microscope. Data was analyzed using ImageJ software (https://imagej.nih.gov/ij/download.html). All data statistics analysis was performed using paired Student's *t*‐tests. The *p* value <0.05 was considered as significant difference exists. * represents 0.01 < *p* < 0.05. ** represents 0.001 < *p* < 0.01. *** represents *p* < 0.001. N.S. represents no significant.

## AUTHOR CONTRIBUTIONS

Bo Dong conceived the project. Most experiments were carried out by Yuanyuan Fu with the help from Deqing Kong and Yuping Fan. Data were analyzed and discussed by Yuanyuan Fu, Zhiyi Lv, and Bo Dong. The initial draft was drafted by Yuanyuan Fu and Zhiyi Lv. Final version of the manuscript was prepared by Bo Dong.

## CONFLICT OF INTEREST

The authors declare no competing or financial interests.

## Supporting information


**Appendix S1** Supporting Information.

## Data Availability

All data generated or analyzed during this study are included in the manuscript and supporting files.
