# Supplementary material for: High abundance of CDC45 inhibits cell proliferation through elevation of HSPA6
Source: Cell Prolif. 2022 Jun 1;55(7):e13257. doi: 10.1111/cpr.13257 (PMC9251052; doi:10.1111/cpr.13257)
Supplement: Supplementary file 1 — Appendix S1 Supporting Information. [file CPR-55-e13257-s001.docx]

**SUPPLEMENTARY INFORMATION**

**Table 1. Gene ID**

| **GENE NAME** | **GENE ID** |
| --- | --- |
| *Hs*-CDC45 | AAC67521.1 |
| *Mm*-CDC45 | AAC95057.1 |
| *Cr*-CDC45 | BAB00625.1 |
| *Dm*-CDC45 | NP_569880.1 |
| *Ec-*RecJ | AAA62789.1 |
| *Hs*-HSPA6 | NM_002155.5 |

**Table 2. PCR Primers**

| **NAME** | **SEQUENCE** |
| --- | --- |
| Hs-CDC45-pEGFP-C1-F1 | CGCGCGAATTCTATGTTCGTGTCCGATTTCCGCAAAGAGTTC |
| Hs-CDC45-pEGFP-C1-R1 | CGCGCCCCGGGCTAGGACAGGAGGGAAATAAGTGCGTCCAG |
| Mus-CDC45-pEGFP-C1-F1 | CGGCTCGAGCTATGTTCGTGACCGATTTCCGCAAGGAGTTC |
| Mus-CDC45-pEGFP-C1-R1 | CGGGGATCCTCAGGACAGCAGTGACACAAGAGCGTCCAG |
| Ci-CDC45-pEGFP-C1-F1 | CGGCTCGAGCTATGTTAATTACCGACCCAGTAAAGGATTTT |
| Ci-CDC45-pEGFP-C1-R1 | CGGGGATCCTTATGACATTATAGTGATGAGAGCGTCAAT |
| Ci-MCM3-pEGFP-N1-F1 | CGCGCGCTAGCATGGCGGAGGTTGAGATTGAACCAGAGCTT |
| Ci-MCM3-pEGFP-N1-R1 | CGCGCCCCGGGATATAAGAAACACAACATCATCGGAAACCAT |
| Hs-MCM3-pEGFP-N1-F1 | CGCGCGCTAGCATGCTTATATGCTCCCAAGGTCCCCGCCTC |
| Hs-MCM3-pEGFP-N1-R1 | CGCGCCCCGGGAGATGAGGAAGATGATGCCCTCAGACACCAT |
| Epi I-CDC45-R1 | TCTAGAGTCGCGGCCGCTTATGACATTATAGTGATGAGAGCG |
| Epi I-CDC45-F1 | CGGTACCGCGGGCCCGGGCAGAAAAAATGGTGAGCAAGGGCG  AGGAGCTGT |
| CMV-Hs-HSPA6-F1 | CACGCGTCGACATGCAGGCCCCACGGGAGCTCG |
| CMV-Hs-HSPA6-R1 | CCCGGGGTACCGCATCAACCTCCTCAATGATGGGGCC |
| Hs-ACTB-qF1 | TCCTCCCTGGAGAAGAGCTA |
| Hs-ACTB-qR1 | CCAGACAGCACTGTGTTGGC |
| Hs-HSPA6-qF1 | TTCGATGCCAAGCGGCTGAT |
| Hs-HSPA6-qR1 | GAACGTCTTGTCCTCCCCGC |
| HSPA6-siRNA1 | GCACAGGUAAGGCUAACAA |
| HSPA6-siRNA2 | CCCGCCUAUUUCAAUGACU |
| HSPA6-siRNA3 | GAGCUGAACAAGAGCAUCA |

**Table3. Reagents**

| **REGENT OR RESOURCE** | **SOURCE** | **IDENTIFIER** |
| --- | --- | --- |
| Anti-GAPDH Mouse Monoclonal Antibody | Transgen | CAT# HC301-01 |
| Anti-GFP Rabbit Monoclonal Antibody | Cell Signaling Technology | CAT# 2956 |
| Phospho-Histone H2A.X (Ser139) Antibody | Cell Signaling Technology | CAT# 2577 |
| Goat Anti-Mouse IgG (H+L), HRP Conjugate | Transgen | CAT# HS201-01 |
| Goat Anti-Rabbit IgG (H+L), HRP Conjugate | Transgen | CAT# HS101-01 |
| Alexa Fluor 568 anti-Rabbit IgG | Invitrogen | CAT# A11011 |
| Vectashield Antifade mounting medium with DAPI | Vector Laboratories | CAT# H-1200 |
| Mouse anti-BrdU | Invitrogen | CAT# B35128 |
| BrdU (5-Bromo-2´-Deoxyuridine) | Invitrogen | CAT# B23151 |
| Anti-PH3 Rabbit Monoclonal Antibody | From Gao’s Lab | N/A |
| Cell Cycle and Apoptosis Analysis Kit | Beyotime | CAT#C1052 |
| Lipofectamine® 3000 | Invitrogen | CAT#L3000015 |
| Cdc45 (D7G6) Rabbit mAb | Cell Signaling Technology | CAT#11881 |
| Phanta Max Super-Fidelity DNA Polymerase | Vazyme | CAT# P505-d1 |
| GeneJET Gel Extration Kit | Thermo | CAT#K0692 |
| T4 DNA Ligase | Clontech | CAT#2011A |
| EndoFree Mini Plasmid Kit II | TIANGEN | CAT# DP118 |
| Western Lightning^®^ Plus-ECL | PerkinElmer | CAT#NEL105001EA |
| HiScript III RT SuperMix for qPCR (+gDNA wiper) | Vazyme | CAT#R323 |
| ChamQ SYBR Color qPCR Master Mix | Vazyme | CAT#Q411-02 |
| TUNEL BrightRed Apoptosis Detection Kit | Vazyme | CAT#A113 |
| Genopure Plasmid Maxi Kit | Roche | CAT#03143422001 |


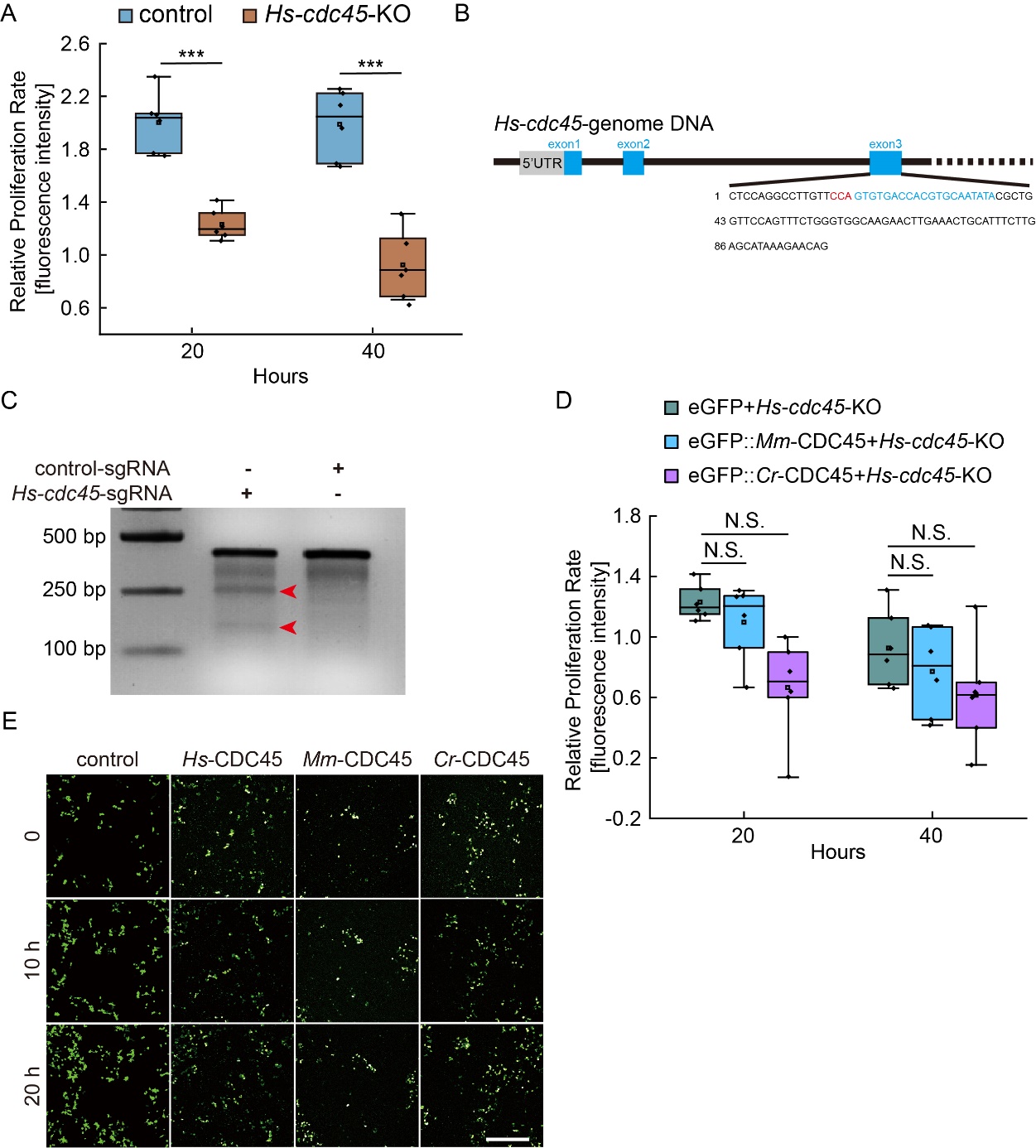


**SUPPLEMENTARY FIGURE 1.** Knockout of *Hs-cdc45* inhibits HEK293T cells’ proliferation rate. (A) Deletion of *Hs-cdc45* induced decrease of cell proliferation rate in HEK293T cells. n = 6. *** *P* < 0.001 by Students *t*-test. (B) Single guide RNA target for *Hs-cdc45* locates onto the third exon of the genome sequence. (C) Two bands with different size (150 bp and 250 bp) showed in the result of T7 endonuclease I cleavage to confirm that *Hs-cdc45* was deleted in HEK293T cells by CRISPR/Cas9. (D) Ectopic expression of *Mm-cdc45* or *Cr-cdc45* could not rescue the cell proliferation rate inhibition induced by *Hs-cdc45* deletion. N.S., No significant. n = 6. N.S. by Students *t*-test. (E) Representative images of *Hs-cdc45*, *Mm-cdc45* and *Cr-cdc45* overexpression inhibiting HEK293T cell proliferation rate, respectively. The images were taken by confocal microscopy at the same position of the 24-well plate at different time points. Scale bar represents 500 μm.


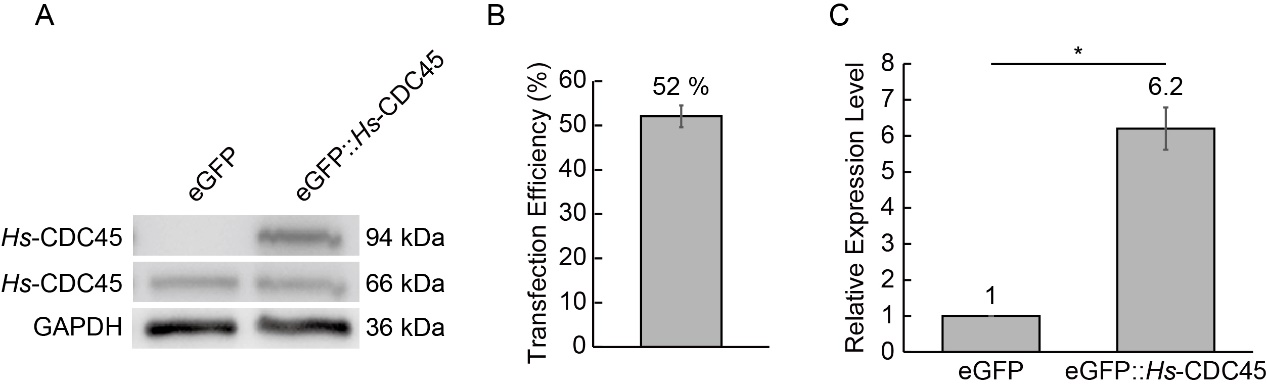


**SUPPLEMENTARY FIGURE 2.** The examination of the protein level of CDC45 overexpression. (A) Representative image of Western Blot to confirm that eGFP::*Hs-*CDC45 was overexpressed in HEK293T cells. (B) The transfection efficiency of HEK293T cells was about 52 ± 2.5 %. Error bar represents standard deviation. n = 3. (C) The level of CDC45 overexpression is about 6.2 ± 0.6 times higher compared to the control group. Error bars represent standard deviation. n = 3. * *P* < 0.05 by Students *t*-test.


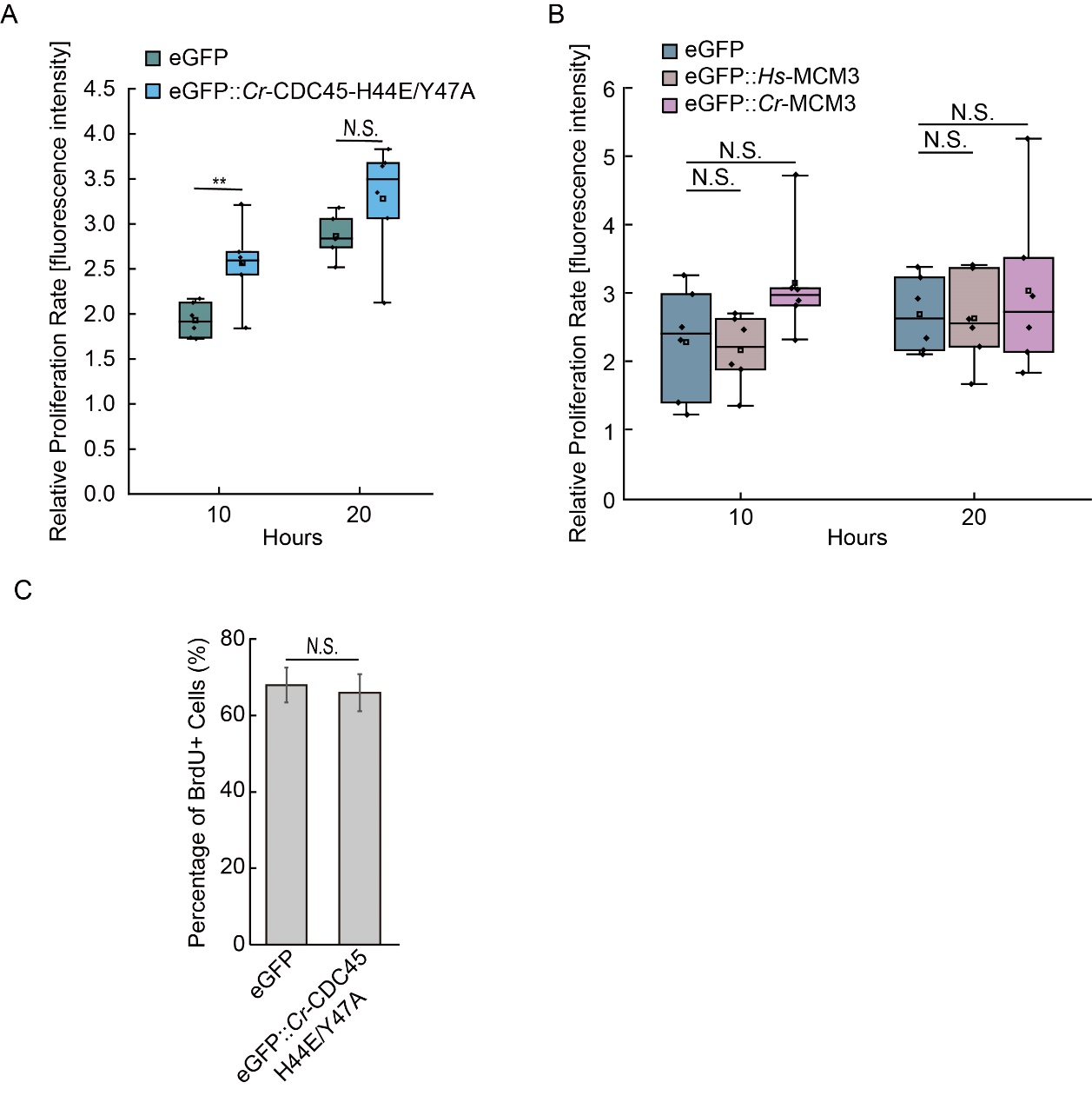


**SUPPLEMENTARY FIGURE 3.** (A) Overexpression of *Cr*-*cdc45*-H44E/Y47A has no negative effect on cell proliferation rate. N.S., No significant. n = 6. N.S. and ** *P* < 0.01 by Students *t*-test. (B) Overexpression of either *Hs-mcm3* or *Cr-mcm3* has no effect on cell proliferation rate. N.S., No significant. n = 6. N.S. by Students *t*-test. (C) Overexpression of *Cr*-*cdc45*-H44E/Y47A has no negative effect on BrdU incorporation into cells. 150 cells per treatment were analyzed. Error bars represent standard deviation. n = 3. N.S., No Significant. N.S. by Students *t*-test.


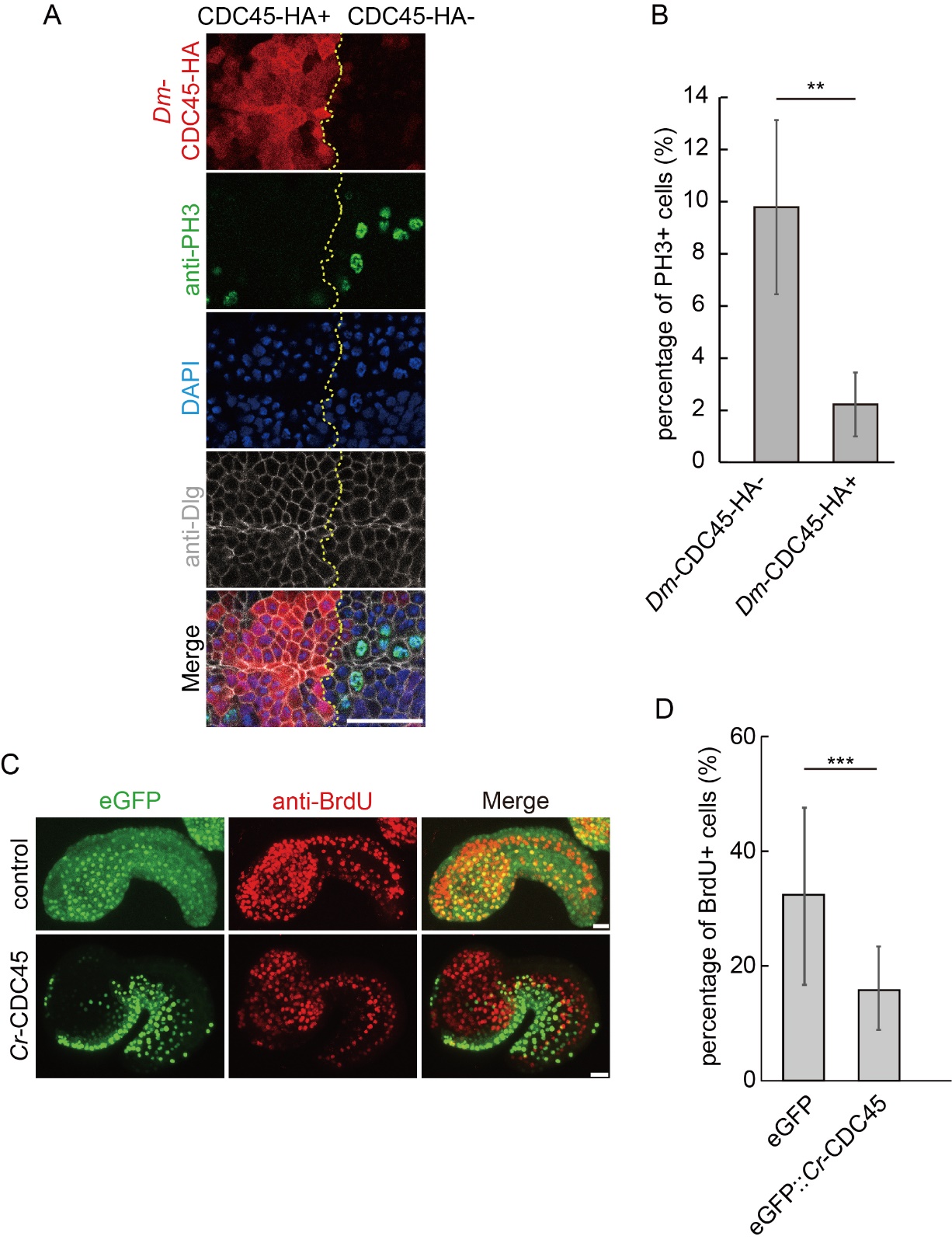


**SUPPLEMENTARY FIGURE 4.** (A) Representative image of PH3 staining of *Drosophila* epidermis cells overexpressing *Dm-cdc45*. (B) Ectopic expression of *Dm-cdc45* induced decrease of PH3 positive signals in *Drosophila* epidermis cells. Error bars represent standard deviation. n = 5. ** *P* < 0.01 by Students *t*-test. (C) Representative image of BrdU incorporation of *Ciona* epidermis cells overexpressing *Cr-cdc45*. (D) Ectopic expression of *Cr-cdc45* induced decrease of BrdU incorporation into *Ciona* epidermis cells. Error bars represent standard deviation. n = 24. *** *P* < 0.001 by Students *t*-test. Scale bar represents 20 μm.


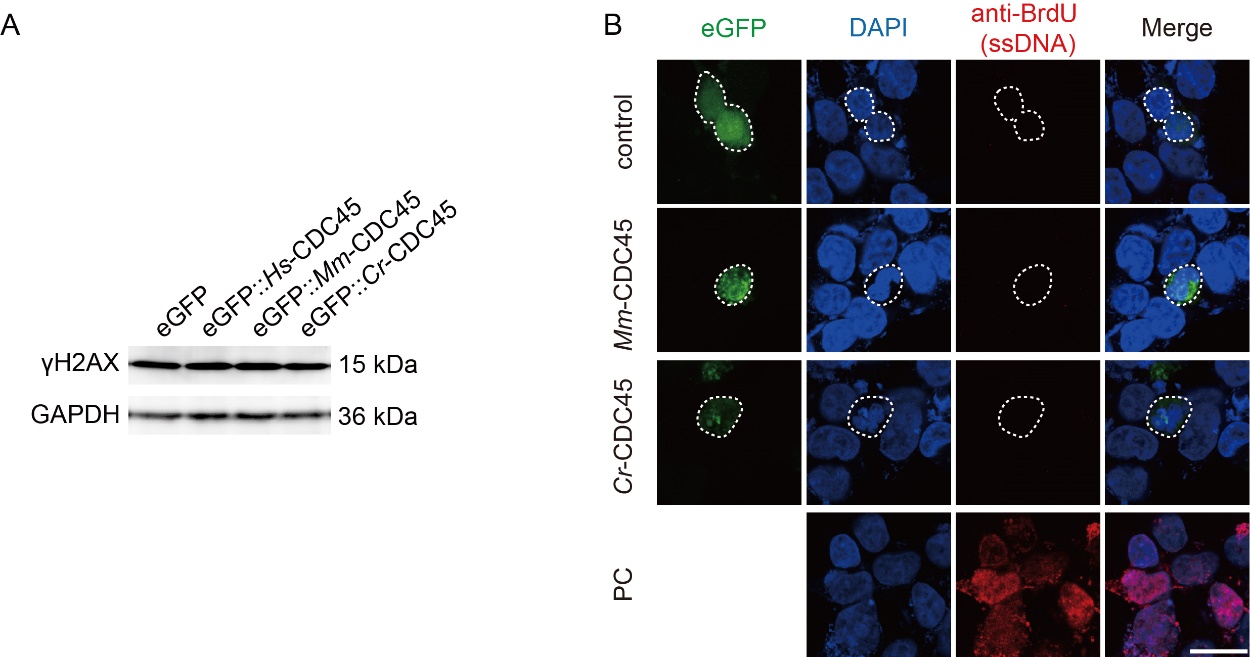


**SUPPLEMENTARY FIGURE 5.** DNA damage detection in the cells overexpressing *cdc45*. (A) No more γH2AX accumulation in HeLa cells overexpressing *cdc45* more than the control group post-transfection 72 hours. (B) No ssDNA detected in HEK293T cells overexpressing of *cdc45.* After one cell cycle in medium supplemented with BrdU, cells were then transfected with vectors encoding eGFP or eGFP::CDC45. ssDNA was visualized by immunofluorescence against BrdU without adding HCl to denature dsDNA step. PC, Positive Control. Scale bar represents 20 μm.


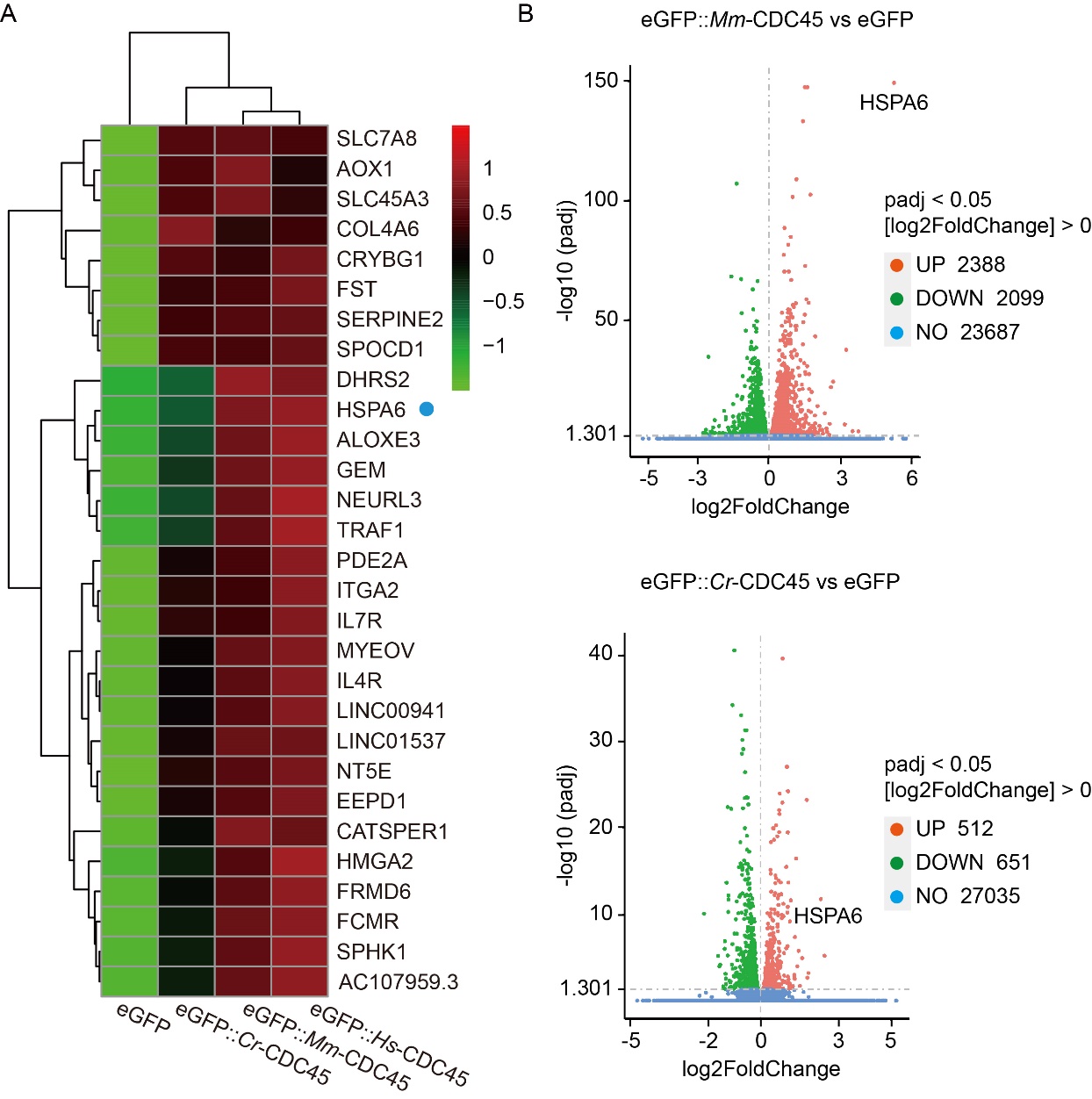


**SUPPLEMENTARY FIGURE 6.** (A) Clustering of differential genes that up-regulated from RNA-seq data that with and without *cdc45* overexpression in HeLa cells (log2FoldChange of *Cr-cdc45* > 1). Red indicates highly expressed genes, whereas green indicates lower expressed genes. Blue dot indicates target gene *hspa6* screened for the following experiments. (B) Volcano plot of differentially expressed genes in HeLa cells overexpressing *Mm-cdc45* and *Cr-cdc45* compared to the control, respectively. *hspa6* was the most up-regulated gene in HeLa cells overexpressing *cdc45*.


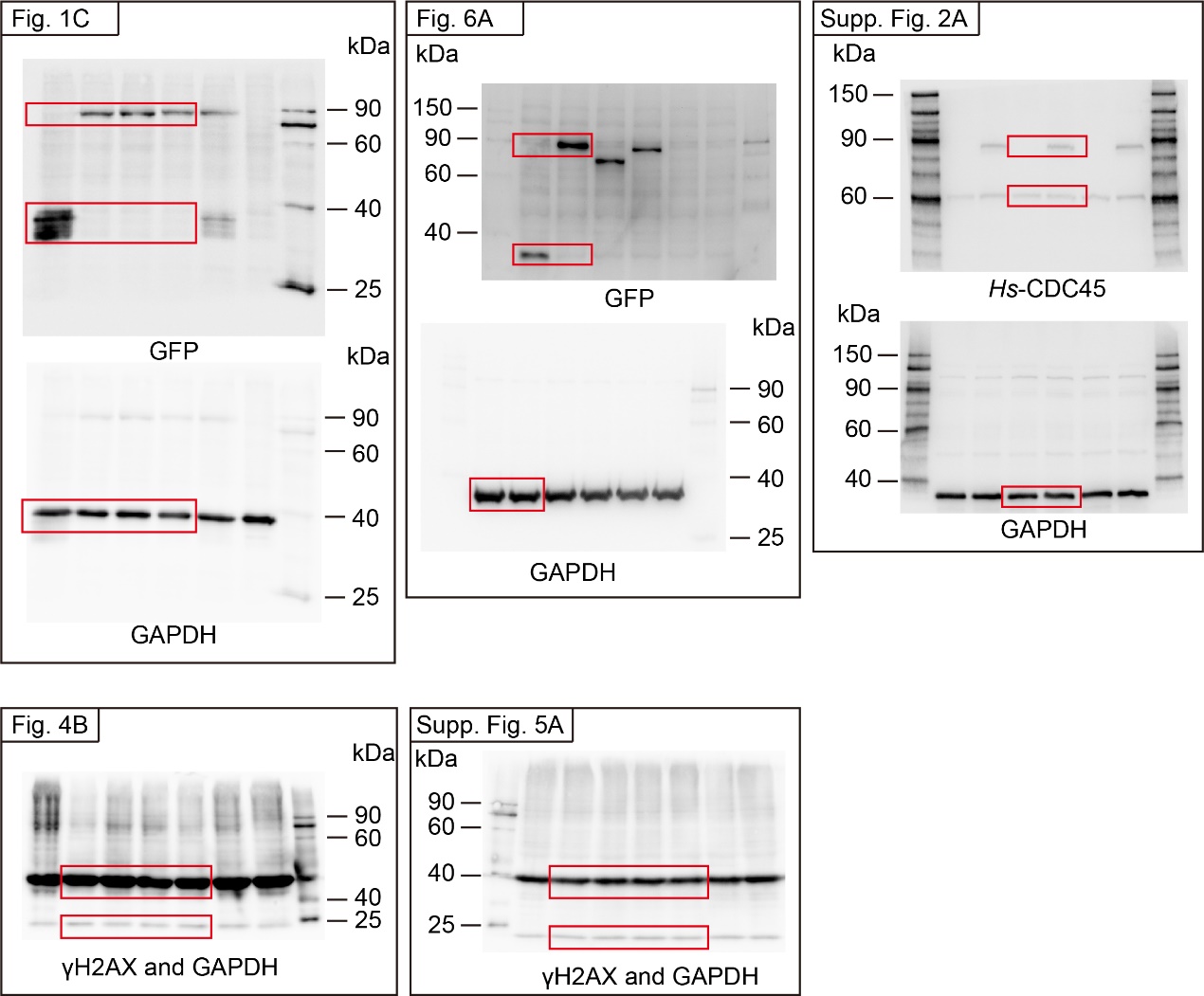


**SUPPLEMENTARY FIGURE 7.** Original data of Figure 1C, Figure 4B, Figure 6, Supplementary Figure 2A and Supplementary Figure 5A.
